# Efficacy of New Generation Antidepressants: Differences Seem Illusory

**DOI:** 10.1371/journal.pone.0063509

**Published:** 2013-06-03

**Authors:** A. C. Del Re, Glen I. Spielmans, Christoph Flückiger, Bruce E. Wampold

**Affiliations:** 1 VA Palo Alto Health Care System, Palo Alto, California, United States of America; 2 Stanford University Medical School, Stanford, California, United States of America; 3 Metropolitan State University, St. Paul, Minnesota, United States of America; 4 University of Zurich, Zurich, Switzerland; 5 University of Wisconsin-Madison, Madison, Wisconsin, United States of America; 6 Research Institute, Modum Bad Psychiatric Center, Vikersund, Norway; Copenhagen University Hospital, Denmark

## Abstract

**Background:**

Recently, Cipriani and colleagues examined the relative efficacy of 12 new-generation antidepressants on major depression using network meta-analytic methods. They found that some of these medications outperformed others in patient response to treatment. However, several methodological criticisms have been raised about network meta-analysis and Cipriani's analysis in particular which creates the concern that the stated superiority of some antidepressants relative to others may be unwarranted.

**Materials and Methods:**

A Monte Carlo simulation was conducted which involved replicating Cipriani's network meta-analysis under the null hypothesis (i.e., no true differences between antidepressants). The following simulation strategy was implemented: (1) 1000 simulations were generated under the null hypothesis (i.e., under the assumption that there were no differences among the 12 antidepressants), (2) each of the 1000 simulations were network meta-analyzed, and (3) the total number of false positive results from the network meta-analyses were calculated.

**Findings:**

Greater than 7 times out of 10, the network meta-analysis resulted in one or more comparisons that indicated the superiority of at least one antidepressant when no such true differences among them existed.

**Interpretation:**

Based on our simulation study, the results indicated that under identical conditions to those of the 117 RCTs with 236 treatment arms contained in Cipriani et al.'s meta-analysis, one or more false claims about the relative efficacy of antidepressants will be made over 70% of the time. As others have shown as well, there is little evidence in these trials that any antidepressant is more effective than another. The tendency of network meta-analyses to generate false positive results should be considered when conducting multiple comparison analyses.

## Introduction

Recently, Cipriani and colleagues [1] examined the relative efficacy of 12 new-generation antidepressants on major depression. They applied a random-effects meta-analytic model that used a Bayesian approach [2] (often referred to as network meta-analysis) to examine 117 randomized controlled trials (RCTs) and concluded, “Mirtazapine, escitalopram, venlafaxine, and sertraline were significantly more efficacious than duloxetine ([estimated] odds ratios [OR] 1.39, 1.33, 1.30 and 1.27, respectively), fluoxetine (1.37, 1.32, 1.28, and 1.25, respectively), fluvoxamine (1.41, 1.35, 1.30, and 1.27, respectively), paroxetine (1.35, 1.30, 1.27, and 1.22, respectively), and reboxetine (2.03, 1.95, 1.89, and 1.85, respectively) [and that] reboxetine was significantly less efficacious than all the other antidepressants tested” (pgs. 746). If these results are reliable, this meta-analysis would have important implications for clinical practice.

Determining the relative efficacy of competing treatments for a particular disorder is of critical importance in evidence-based medicine for improving the quality of care and reducing costs. Cipriani et al.'s efforts to use a sophisticated method to determine which antidepressants are more effective than others is commendable. Nevertheless, a number of concerns have been raised with regard to network meta-analysis [3]–[6]. For example, Trinquart, Abbé, and Ravaud [3] showed that reporting bias had a particularly pernicious effect on the results of network meta-analyses because the bias extended to treatments for which there was no bias; in this way the reporting bias of a particular treatment affected the ranking of all treatments, regardless of whether there was bias for the other treatments. Further, in antidepressant research, selective publication of results from placebo-controlled trials has been well-documented [7]. Across the medical literature in general, including head-to-head antidepressant trials, reporting bias occurs frequently [8]–[11]. Indeed, the reported superiority of escitalopram over citalopram reported in Cipriani et al. was partially driven by the exclusion of a head-to-head study in which mean change on the two drugs was nearly identical yet in which response rates were not reported, thus making it ineligible for inclusion in their analysis (study MD-02) [11]. One critical letter to the editor [10] suggested that “Meta-analysis of published plus industry-furnished data could spuriously suggest that the best drugs are those with the most shamelessly biased data.” (pgs. 1759–1760).

Another critical issue with regard to network meta-analysis is that statistically significant differences detected among pairs of treatments produced by this type of meta-analyses may have a high probability of occurring by chance [5]. Recently, Wampold and Serlin [5] examined two statistical models for testing the null hypothesis that the true difference between any pair of treatments from a set of treatments is zero (i.e., a null of no treatment differences among a set of *k* treatments) and found that both models appropriately protected error rates and were adequately powered to detect alternative hypotheses for rather small effects under various scenarios. When these methods were used to test the null hypothesis that there were no differences among the 12 antidepressants using Cipriani et al.'s [1] data, Wampold and Serlin found that there was insufficient evidence to reject the null that any of the antidepressants was more effective than any other. Given k = 12 antidepressants, there are k(k−1)/2 = 66 pairwise comparison and it appears that the observed differences among the antidepressants may have occurred by chance and were not due to systematic differences among the antidepressants, a result that is consistent with other analyses of the same antidepressant trials [12] but inconsistent with Cipriaini et al.'s conclusion. In fact, the size of the effects produced by the RCTs were considerably less that would be expected under the null hypothesis of no differences [5].

The possibility that the statistically significant differences among the antidepressants found by Cipriani et al. were spurious creates the concern that the stated superiority of some antidepressants relative to others is unwarranted. Indeed, a recent network meta-analysis failed to replicate the results of Cipriani et al. [12]. This may relate to Cipriani et al. only including comparative antidepressant trials in their analyses whereas Gartlehner et al. also included placebo-controlled trials, or alternatively, the discrepancy may be due to spurious findings.

The purpose of the present study was to conduct a Monte Carlo study that mimicked the data from Cipriani et al. under the null of no antidepressants differences to determine how likely it is produce one or more spurious results.

## Materials and Methods

A Monte Carlo simulation was conducted that involved replicating Cipriani's network meta-analysis under the null hypothesis (i.e., no true differences between antidepressants). An overview of the simulation strategy is as follows: (1) 1000 simulations were generated under the null hypothesis of no differences among the 12 antidepressants, (2) each of the 1000 simulations were network meta-analyzed, (3) the total number of false positive results from the network meta-analyses were calculated.

One-thousand simulated datasets were generated that were equivalent to Cipriani's dataset in all aspects (viz., sample size, antidepressants, and treatment comparisons). The response for each participant in each study was randomly generated under a null hypothesis of no differences in which the probability of a positive response was equal to the response rate for the total sample reported in Cipriani et al. The studies in Cipriani's meta-analysis included 25,928 participants, of whom 0.57 had a positive response to treatment. Consequently, in each of the simulations, the probability that an individual participant, regardless of arm in the study, would have a positive response was set at 0.57 prior to running the simulations. A 1 was assigned for a positive response, and 0 otherwise, such that P(response  = 1)  = 0. 57. Then the response of all participants in the 117 trials were randomly repeated 1000 times under the null hypothesis that the probability of a response was not conditioned on the antidepressant administered.

The simulated data were then analyzed with network meta-analysis using the ‘gemtc’ network meta-analysis package in the R statistical software program [13]. Odds Ratios (OR) were calculated from the resulting logistic point estimates, along with the log odds of the standard errors. Before conducting the simulation study, the software was validated by analyzing the results of the 117 RCTs and we found that the results were in accord with Cipriani et al. at a level of precision of ±0.01. For each of the 1000 simulations, the OR as well the standard error for each of the k(k−1)/2 = 66 comparisons between pairs of the k = 12 antidepressants were used to determine the statistical significance of the pairwise comparisons: A false positive occurred when the 95% confidence interval for the OR *did not* include 1, indicating that one antidepressant was considered to be superior to the comparison.

## Results

**Figure 1:**
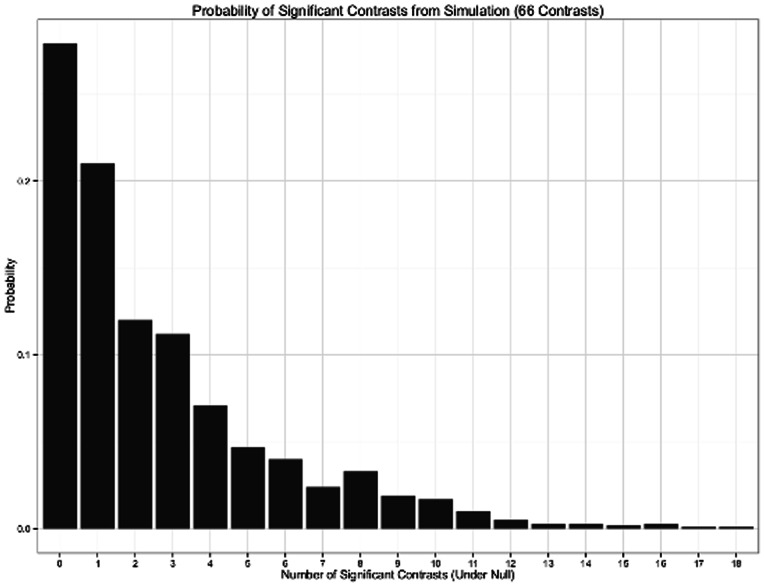
Probability of Significant Contrasts from Simulation (66 Contrasts). The *y*-axis represents the probability of significant contrasts. The *x*-axis represents the number of significant contrasts for each of the 1000 network meta-analytic replications, each with 66 contrasts.

For each of the 1000 replications, let *x* be the number of false positives (i.e., falsely concluding that antidepressant A was superior to antidepressant B based on α = 0.05) obtained for that replication. The values of *x* range from 0 (for that replication, there were no statistically significant differences) to 66 (all pairwise comparisons for the replication yielded statistical significance). A frequency f(x) for each value of *x* was constructed to indicate the number of replications that produced that value of *x*. The probability distribution X was determined by computing f(*x*)/1000 for each value of *x*. That is, P(X = *x*)  =  f(*x*)/1000, which is the probability of obtaining *x* number of significant comparisons. If network meta-analysis maintains the proper family-wise error rates, the probability of obtaining one or more false positives would be less than 0.05 (i.e., 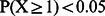). The probability distribution of X is shown in Figure 1, truncated at X = 18. For this distribution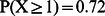; thus, in more than 7 times out of 10, the network meta-analysis resulted in one or more comparisons that indicated the superiority of at least one antidepressant when no such true differences among them existed.

The expectation of the probability distribution of X was equal to 2.68 (i.e, 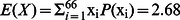). On average, it is expected that 2.68 significant differences will be detected by chance. Interestingly, if the 66 comparisons were conducted independently as a binomially distributed random variable with parameters *n* = 66 comparisons and probability of false rejection of *p* = 0.05, the expectation would be *np*  = 3.30. Consequently, network meta-analysis offers some protection, in that the expected number of false positives was reduced from 3.30 to 2.68. However, the relative reduction is not comforting as there remains a probability of 0.72 of detecting one or more differences by chance.

## Discussion

Based on the results of their network meta-analysis, Cipriani et al. [1] concluded, “Our findings might help to choose among new-generation antidepressants” (p. 753). Conclusions about the relative efficacy of medical antidepressants should only be made if the null hypothesis of no treatment differences is rejected, conventionally based on an error rate of 0.05. Based on our simulation study, the results indicated that under identical conditions to those of the 117 RCTs with 236 treatment arms contained in Cipriani et al.'s meta-analysis, one or more false claims about efficacy will be made over 70% of the time. As others [5], [6], [12] have shown, the pattern of results in the 117 RCTs are consistent with the null hypothesis that the 12 antidepressants are equally efficacious, so the findings of those analyses and our simulation suggest that Cipriani et al.'s conclusions should not be relied upon to make clinical decisions.

The results of the simulation study are specific to the parameters of the RCTs in the Cipriani meta-analysis in terms of number of trials, number of participants per trial, and response rate. Consequently, network meta-analyses may perform differently under different scenarios. However, it is clear that the use of network meta-analysis to examine all *k*(*k*−1)/2 pairwise comparisons of *k* treatments with conventional alpha levels is problematic. The implications of conducting multiple statistical tests on the family-wise error rates are well known [14].

If one is interested in the comparison of two particular treatments, say Treatment A and Treatment B, and there are insufficient number of trials comparing A and B, then the use of a method that increases the power of the comparison by incorporating information from indirect paths, in the way that network meta-analysis does, may be warranted. In that way, the method is used to test a focused hypothesis with a powerful meta-analytic strategy [15]–[17]. One of the advantages of Bayesian MTC methods over conventional meta-analysis is that one can examine and take into consideration the likelihood of the alternative hypothesis (i.e., estimate the probability that a treatment should be preferred). For example, conducting a rank probability test using MTC methods will estimate the probability of each treatment to be superior to other treatments (i.e., the probability of treatment A ranking first, ranking 2nd, etc and the probability of treatment B ranking first, and so on).

If one wanted to extend the method to compare one treatment, say treatment A, to various other treatments, say B, C, and D, then one could compare A to the effects of B, C, and D pooled, although the details of how this would work in the context of network meta-analysis has not yet been derived. In the antidepressant trials reviewed in Cipriani et al., reboxetine appears to be inferior to other antidepressants, a result that has been meta-analytically examined and confirmed [9]. If this is the conjecture of interest, then the analysis should be focused on the relative efficacy of reboxetine versus other antidepressants. Indeed, we examined the 8 trials in Cipriani et al. that directly compared reboxetine to one of the other 12 antidepressants and conducted a standard meta-analysis. A restricted maximum likelihood univariate meta-analysis [18]–[20] of these 8 trials reveals that reboxetine was inferior to the other antidepressants (OR  = 0.81, [0.68, 0.98], p = .03), suggesting in this case that utilizing the indirect paths was not needed to reach this conclusion and emphasizing the importance of using statistical power to focus on a particular conjecture.

If one wishes to consider all pairwise comparisons, as Cipriani et al. did, family-wise error rates must be considered. One way to control the family-wise error rate would be to use a Bonferroni correction for Type I error, although this is a fairly conservative approach [21]. The Bonferroni correction involves altering the alpha-level based on the total number of comparisons by dividing the alpha by the number of comparisons. For example, in the present case, 66 pairwise comparisons with an alpha of 0.05 yields a Bonferroni correction of 0.05/66 = 0.0008 for each comparison, although less conservative options might be available [21]. However, in the case of Cipriani et al., it is critical to keep in mind that the omnibus test of the null hypothesis of no true differences among any of the antidepressants was not rejected, making the search for pairwise differences unwise [5].

The purpose of network meta-analysis is to identify which antidepressants from a set of antidepressants are more effective than others. We have shown in one case that caution must be used when applying this method, as the probability of false positive is unacceptably high. Such analyses do not address whether the set of antidepressants is effective, evidence for which would be established by conventional meta-analyses of the antidepressants versus placebo controls.

## Conclusion

The results of this simulation indicated that under identical conditions to those of Cipriani et al.'s meta-analysis, one or more false claims about efficacy will be made 71% of the time. As other research has shown, the pattern of results in the 117 RCTs is consistent with the null hypothesis that the 12 antidepressants are equally efficacious. The findings of those analyses and our simulation suggest that Cipriani et al.'s conclusions should not be relied upon to make clinical decisions, although the inferiority of reboxetine was confirmed by a meta-analysis of direct comparisons.

## Supporting Information

Appendix S1 **R code to Replicate Output of MTC Meta-Analysis.**
(R) Click here for additional data file.
